# Bone Fracture Incidence After Androgen Deprivation Therapy-Investigational Agents: Results From Cancer Therapy Evaluation Program-Sponsored Early Phase Clinical Trials 2006–2013

**DOI:** 10.3389/fonc.2020.01125

**Published:** 2020-07-14

**Authors:** Zin W. Myint, Charles A. Kunos

**Affiliations:** ^1^Division of Medical Oncology, Department of Internal Medicine, University of Kentucky, Lexington, KY, United States; ^2^Cancer Therapy Evaluation Program, National Cancer Institute, Bethesda, MD, United States

**Keywords:** fracture risk, ADT, prostate cancer, bone mineral density, comorbidities

## Abstract

**Introduction:** Androgen deprivation therapy (ADT) is a primary treatment option for patients diagnosed with locally advanced-stage or metastatic prostate cancer. Androgen deprivation can be achieved either by radical orchiectomy or by medical castration using a gonadotropin-releasing hormone agonist. ADT has been linked to an initial 12-month loss of bone mineral density, a risk factor for weight-bearing bone fracture, and therefore, a confounding hazard for adverse event when patients are enrolled on early phase trials. To better understand the frequency of ADT-investigational agent-related bone fracture, we conducted a retrospective study of National Cancer Institute Cancer Therapy Evaluation Program (CTEP)-sponsored early phase trials to determine the number of fractures observed among enrolled prostate cancer patients.

**Patients and Methods:** 464 locally advanced-stage or metastatic prostate cancer patients were identified among seven ADT-investigational agent trials conducted between 2006 and 2013. Demographic, co-morbidity, treatment, and adverse event variables were abstracted from CTEP databases and descriptive statistics were used.

**Results:** 464 men had a median age of 64 years, were mostly white (90%), and had a performance status of 0 or 1 (98%). The number of new bone fractures occurring on or after ADT-investigational agent treatment was very low (4.6 per 1000 person-years). The median pretrial prostate specific antigen level was 29 ng/mL and most men (71%) had prostate cancer histopathology Gleason 7 score or higher. In these trials, 43 percent of men had bone only and 35 percent had bone and visceral metastatic disease. The most frequent grade 1 or 2 adverse events were fatigue (36%), hot flashes (27%), and anemia (17%). Grade 3 or higher adverse events were rare, with hypertension (3%) and hyperglycemia (3%) observed.

**Conclusions:** Identifying bone health factors may still be relevant in selected early phase ADT-investigational agent trial patients, emphasizing the need for improved methods for capturing baseline bone health and studying ADT-investigational agent and concurrent medication interactions on bone health.

## Introduction

Androgen deprivation therapy (ADT) is one of the main treatments for both locally advanced-stage and metastatic prostate cancer (1, 2). It can take the form of either radical orchiectomy or medical castration by gonadotropin-releasing hormone agonist (GnRH; or luteinizing hormone-releasing hormone [LHRH]) therapy. ADT is not benign, as it is associated with rapid loss of bone mineral density within the first 12 months of initial therapy, a hazard for weight-bearing bone fracture (3, 4).

In 2005, a Surveillance, Epidemiology, and End Results (SEER) Program/Medicare claims study of over 50,000 5-year prostate cancer survivors found that those who received ADT had a significantly higher risk of fracture compared to those who did not receive ADT (19 vs. 13%, respectively; *p* < 0.001) (5). More studies also have supported an association between ADT exposure and a risk for subsequent bone fracture (6–8). In some retrospective studies of ADT-treated men with prostate cancer, medical co-morbidities like diabetes, autoimmune disease, and liver disease exacerbated a risk for bone fracture (9, 10). Concomitant protracted corticosteroid use also elevates the risk for bone fracture (7). It is also established that single or multiple hip, vertebral, or appendicular long-bone fractures substantially interferes with quality of life (11). All of these factors render the full health impact of ADT-investigational agent treatment important to early phase trials wherein bone health might be affected.

To better understand any impact of ADT-investigational agent treatment upon bone health, we undertook a retrospective study of United States National Cancer Institute Cancer Therapy Evaluation Program (CTEP)-sponsored early phase trials to tabulate the number of bone fractures observed on or after trial ADT-investigational agent treatment among enrolled men with prostate cancer.

## Patients and Methods

Eligible study subjects were found by computer database search (iPAD, version 6.5.1) of CTEP-sponsored early phase trials involving investigational agents that permitted concurrent use of ADT (**Figure 1**). Between 2006 and 2013, seven trials met criteria for retrospective review [NCI S0925, (12) E2809, (13) 7640, (14) 7864 [NCT00589472], 8014, (15) 8384 [NCT01163084], and E9802 (16)]. Demographic, co-morbidity, treatment, and adverse event variables were abstracted. Enrolled subjects sustaining fractures on trial treatment had additional variables abstracted, such as grade of fracture or hospitalization, if any. Clinicopathological data including baseline median prostate-specific antigen (PSA) level, histopathological Gleason score, race, Eastern Cooperative Oncology Group (ECOG) performance status, baseline disease site (bone only, node only, visceral only, or multiple sites), prior therapy (hormonal, surgical castration, combined androgen blockade with prior bicalutamide, radiation), fracture (baseline or on-treatment), hospitalization if any, and other reported adverse events were collected from computer databases. Concurrent medications, such as concomitant use of bone health agents, taken by participants on trial was not accessible. Descriptive statistics were used in this analysis; advanced statistical analyses were considered moot given the very low incidence of new bone fractures found in review. Deidentified variables were collected and no subjects were contacted; and thus, this study is considered institutional review board exempt.

## Results

Total seven CTEP-sponsored early phase trials of men with prostate cancer treated by ADT-investigational agent therapy were identified between 2006 and 2013. The seven trial evaluated the investigational agents cixutumumab, MK2206, pazopanib, vorinostat, AT-101, vismodegib, or a pox virus vaccine; trial data are reported elsewhere (12–16). Together, the trials enrolled 464 patients with the median duration of treatment was 30 weeks [95% CI (13–46)] and the median follow up was 33 weeks [95% CI (17–82)] (Table 1). The median age was 64 years in our 464 patients and the majority were white (90%, Table 2). ECOG performance status was either 0 or 1 in almost all men on trial (98%). Taken together, 43 percent of men had bone only and 35 percent had bone and visceral metastatic disease. Median baseline PSA level at trial enrollment was 29.3 ng/ml. Prior therapies for these men included hormonal therapy (36%), surgical castration (37%), and any type radiotherapy (16%). Most had a histopathological Gleason score of 7 or higher (71%). The duration of ADT-investigational agent exposure ranged from 6 weeks to 72 weeks.

**Table 1:** Description of the 7 NCI early phase trials included in this study.

**NCI protocol #**	**Trial activation date**	**Study description**	**Study population**	**Duration of treatment (weeks) (95%CI)**	**Median follow-up duration (weeks) (95%CI)**	**References**
0925	12/15/2010	Phase II randomized multi-center study Arm 1 – oral bicalutamide daily with LHRH agonist + cixutumumab 10 mg/kg IV over 1 h every 2 weeks for 7 cycles (each cycle = 2 treatments in 28 days) Arm 2 – bicalutamide with LHRH agonist	Metastatic hormone sensitive prostate cancer (mHSPC)	28 (13–46)	24 (17–82)	(12)
2809	12/23/2010	Randomized phase II study Arm A – cycles 1- 3 observation; cycles 4-11 bicalutamide 50 mg daily Arm B – cycles 1–3 MK2206 200 mg once weekly orally; cycles 4-11 MK2206 + bicalutamide	N0M0 non-castrate biochemical recurrence (Rising PSA population)	44 (13–46)	102 (17–82)	(13)
7640	09/06/2007	Phase II multi-institutional study Arm A – Pazopanib Arm B – Pazopanib + bicalutamide 50 mg daily on day 8 of cycle	Chemo-naïve castration resistant prostate cancer (CRPC)	Until disease progression(13–46)	8 (17–82) (terminated early due to slow accrual)	(14)
7864	11/29/2007	Neoadjuvant study Bicalutamide for 1 month and leuprolide acetate IM or goserelin acetate subcutaneously once a month until surgery. Vorinostat oral beginning on the first day of ADT and continuing up to 8 weeks or until the day of surgery. Patients then undergo an open or laparoscopic radical prostatectomy. Patients with positive surgical margins undergo immediate adjuvant external beam radiotherapy to the prostatic fossa.	Localized prostate cancer	8 (13–46)	27 (17–82)	No published data
8014	04/04/2008	Phase 2 multi-center study LHRH agonist + bicalutamide (30 days during the first month of LHRH agonist) + AT-101 20 mg/day on days 1 to 21 of a 28-day cycle. Maximum 8 cycles of AT-101 treatment.	mHSPC	32 (13–46)	33 (17–82)	(15)
8384	07/09/2010	Randomized phase Ib/II study Preoperative GDC-0449 (vismodegib) and ADT vs. ADT alone followed by radical prostatectomy for select patients with locally advanced prostate cancer.	Localized prostate cancer	16 (13–46)	70 (17–82)	No published data
9802	02/03/2006	Phase II multicenter single arm study PROSTVAC-V (vaccinia; 2 × 10^8^ pfu) subcutaneously on day 1 with subsequent boosts using PROSTVAC-F (1 × 10^9^ pfu) given subcutaneously on day 1 of weeks 5, 9, 13, 17 and then every 12 weeks until biochemical or clinical progression. At progression, eligible patients (without metastasis) were offered bicalutamide 50 mg orally every day for 1 month and LHRH agonist	Hormone sensitive non-metastatic prostate cancer	48 (13–46)	82 (17–82)	(16)

**Table 2:** Patient or tumor characteristics and prior therapies.

	**NCI protocol**									
**Characteristic (at baseline)**	**0925 Arm 1**	**0925 Arm 2**	**2809**	**7640**	**7864**	**8014**	**8384**	**9802**	**Total**	**Percent**
Number of Patients	105	105	108	23	18	55	10	40	464	
Median age (years)	65	66	66	71	MD	61	63	62.5	64	
**Race**										**475 patients**
Asian		8	1	3					12	3%
African American		14	9		3	6		4	36	8%
Native Hawaiian		1							1	0.2%
White		183	98	20	15	49	9	45	419	90%
Unknown		5			1		1	1	7	1%
**Performance status**										**283 patients**
ECOG 0	62	65	MD	16	MD	MD	MD	39	182	64%
ECOG 1	41	38	MD	17	MD	MD	MD	1	97	34%
ECOG 2	2	2	MD		MD	MD	MD		4	1%
**Baseline disease site(s)**										**288 patients**
Bone only	56	63		5	MD		MD	MD	124	43%
Node only	15	9		2	MD		MD	MD	26	9%
Visceral only	15	16		2	MD	3	MD	MD	36	13%
Multiple sites	19	17		14	MD	52	MD	MD	102	35%
**PSA**										
**Median PSA level**	31	37	5.1	129	MD	27.6	MD	4.3	29.3	
**Gleason score**										**318 patients**
<7	8	6		MD	MD	MD	MD	MD	14	4%
7	29	11		MD	MD	MD	MD	MD	40	13%
>7	63	82	81	MD	MD	MD	MD	MD	226	71%
**Prior therapy**										**381 patients**^†^
Prior hormonal therapy	59	65		2	MD	MD	MD	13	139	36%
Surgical castration	MD	MD	81	21	MD	MD	MD	39	141	37%
Combined androgen blockade with prior bicalutamide	MD	MD		18	MD	MD	MD	MD	18	5%
Radiation therapy	MD	MD	30	MD	MD	MD	MD	32	62	16%

† *Percentages do not total 100 because therapy modalities are not mutually exclusive. MD, missing data*.

The collective adverse events (AEs) for the included trials are cataloged in Table 3. Most AEs attributed by ADT were grade 1 or 2, with the most common being fatigue (36%), and hot flashes (27%). The AEs most likely attributed by the investigational agents were anemia (17%), hyperglycemia (15%), and increased alanine aminotransferase (14%). The most frequent grade 3 or higher AEs were hypertension (3%) and hyperglycemia (3%).

**Table 3:** Adverse events.

**Protocol #**	**0925**		**2809**		**7640**		**7864**		**8014**		**9802**		**Attributed ADT Exp**		**Total**			
**# Patients**	**210**		**108**		**23**		**18**		**55**		**40**				**454**			
**AE grade group**	**1–2**	**>3**	**1–2**	**>3**	**1–2**	**>3**	**1–2**	**>3**	**1–2**	**>3**	**1–2**	**>3**			**1–2**	**%**	**>3**	**%**
Constitutional	103				16	5	1		36		41	1	✓	✓	197	43	6	1
Dermatology			20								31			✓	51	11		
Endocrine	136	7	11	4	78	14	3		45		4	5	✓	✓	277	61	30	7
Hematology	85	2		5	8	2	17		18		19	1		✓	147	32	10	2
GI	70	3		1	47	3			90		10			✓	217	48	7	2
Renal	5				10		2				1			✓	18	0	1	0
Sexual	14	1					2						✓		16	4	1	0

Bone fractures happening on ADT-investigational agent treatment, or in the posttherapy observation period, were rare in these trials. Seven (7) men reported having a fracture at any time point before, during, or after trial treatment; five (5) had pretherapy unrelated fractures (i.e., they had fractures prior to starting any ADT and the etiology of their underlying fracture was unknown) and only two (2) men developed new fractures while on trial-related ADT-investigational agent treatment. The incidence of new fracture on ADT-investigational agent treatment was 4.6 per 1,000 person-years. Table 4 details each case. The median age for patients with baseline bone fracture was 80 years, none of whom developed new fractures while on ADT. Of the two men who developed new fractures while on ADT-investigational agent treatment, the median age was 68 years. These men reported no falls nor traumas. One (50%) man underwent surgical intervention for his bone fracture.

**Table 4:** Subject case descriptions for those with bone fracture.

**No**	**Age**	**Duration of LHRH agonist**	**Duration of other treatments**	**Duration of median follow-up**	**Fracture time and site**	**Grade (CTCAE v.5.0)**	**Hospitalization**	**Outcome**
1	60	ADT monthly + Bicalutamide daily × 3 months	Cetuximab every 2 weeks × 3 cycles (total 12 weeks)	24 weeks	**On-treatment fracture** right lesser trochanter avulsion fracture and small subchondral fracture of right femoral head	3	Right femur intramedullary nailing	Withdrawn from protocol after 3 cycles due to progression of bone metastases
2	76	Bicalutamide daily × 28 months	Pazopanib daily × 28 months	8 weeks	**On-treatment fracture** after 28 months on ADT	1	No	Received treatment for 35 months
3	84	ADT monthly + Bicalutamide daily × total 7 months	Cetuximab every 2 weeks × 7 cycles (total 28 weeks)	24 weeks	Baseline spinal (prior to starting ADT)	1	No	Finished study protocol
4	60	ADT monthly + Bicalutamide daily × total 7 months	Cetuximab every 2 weeks × 7 cycles (total 28 weeks)	24 weeks	Baseline hip (prior to starting ADT)	3	No	Finished study protocol
5	93	ADT monthly + Bicalutamide daily × total 7 months	Cetuximab every 2 weeks × 7 cycles (total 28 weeks)	24 weeks	Baseline hip and spinal (prior to starting ADT)	2 (hip) 1 (spinal)	No	Finished study protocol
6	80	ADT monthly + Bicalutamide daily × total 5 months	Cetuximab every 2 weeks × 5 cycles (total 20 weeks)	24 weeks	Baseline fracture (prior to starting ADT)	1	No	Received 5 cycles
7	54	Bicalutamide daily (unknown exact duration)	None	8 weeks	Baseline hip (prior to starting ADT)	3	No	Finished protocol

Patient 1 (Table 4) was a 60-year-old man with metastatic prostate cancer enrolled to NCI #0925 and randomly allocated to oral bicalutamide 50 mg daily and leuprolide acetate 22.5 mg every 12 weeks plus cixutumumab 10mg/kg intravenously over 1 h every 2 weeks for seven cycles. He began therapy in January 2012. In April 2012, he developed right lower extremity pain and inability to bear weight with no history of trauma or fall. MRI and CT scans of the right hip and pelvis demonstrated a pathologic avulsion fracture of the right lesser trochanter with displaced osseous fragment measuring ~1.5 cm. There was also a very small subchondral fracture of the right femoral head. He was hospitalized for 1 week and then underwent a right femur intramedullary nailing. He underwent his third treatment cycle without delay. In May 2012, he was hospitalized for hypercalcemia, and during work up, progression of osseous metastases was found. He discontinued trial therapy that same month.

Patient 2 (Table 4) was a 78-year old man with localized prostate cancer in February 1992 who underwent first-line radiotherapy. After a biochemical recurrence in July 1997, he was treated with surgery followed by hormonal therapy. He had spine metastases discovered in September 2012. For treatment, he enrolled in NCI #7640, a phase II study of pazopanib with or without bicalutamide in hormone refractory prostate cancer. He was randomly allocated to the pazopanib and bicalutamide arm. Protocol therapy initiated in October 2012. He developed grade 1 fracture in February 2013, which was attributed to bicalutamide exposure. The patient received a total of 38 cycles of protocol therapy until grade 3 weight loss and electrolyte abnormalities attributed to pazopanib exposure prompted study withdrawal in September 2013.

## Discussion

In this retrospective study, the incidence of new bone fracture occurring after ADT-investigational agent treatment was very low (29 per 1000 person-years). Due to this finding, this study cannot evaluate the full health effect of ADT-investigational agent treatment in early phase trials where bone health is perhaps impacted. Further prospective study is needed.

Most men in this retrospective study received a short duration of ADT in combination with investigational agents–the duration of ADT exposure ranged from only 6 weeks to 72 weeks. Such a short exposure to ADT is unlikely to adversely impact bone health (17). Moreover, the investigational agents in these trials (pazopanib [a tyrosine kinase inhibitor], cixutumumab [an epidermal-growth factor receptor inhibitor], PROSTVAC-V [an immunotherapy vaccine], MK2206 [an inhibitor of the serine/threonine protein kinase Akt], vismodegib [a Hedgehog pathway inhibitor] and AT-101 [an anti-apoptotic bcl2 protein]) are not known to be associated with adverse bone health or bone fractures. While this study cannot disassociate the investigational agent from an adverse bone health affect or alteration in risk for bone fracture, it is unlikely these agents confound our observations of a low bone fracture rate.

In our retrospective study, only two men developed a bone fracture while receiving ADT-investigational agent therapy (4.6 incidence per 1000 person-years). Bicalutamide exposure was common in each case. Patient 1 received only two doses of LHRH agonist and bicalutamide for more than 3 months before developing a grade 3 fracture. Patient 2 developed grade 1 fracture after 28 months of bicalutamide. Due to the rare incidence of bone fracture in these studies, no generalizable findings are discussed.

It is of note that none of the patients who had a baseline fracture went on to develop a new fracture while on trial permitted ADT. While the reasons for their baseline fractures are unknown, based on the elderly age of the patients (median 80 years) it is likely that co-morbid osteopenia is a confounding factor (18). Firm conclusions cannot be made here.

ADT exposure varied from 3 to 28 months. It is well-known that ADT causes loss of bone mineral density (BMD), and longer treatment is associated with more rapid loss. BMD decreases by two to eight percent in the first year of ADT initiation (19, 20). A 2018 study used dual energy X-ray absorptiometry (DEXA) every 6 months for a 2-year period to evaluate change in lumbar BMD of Japanese prostate cancer patients on ADT for 2 years. This study found that lumbar BMD decreased by 4% at 1 year and by 6% at 2 years (21). A multi-center prospective observational study of Asian men with prostate cancer was performed in 2017. These patients were treated with either continuous combined ADT vs. GnRH agonist monotherapy and were followed with DEXA for their BMD and Fracture Risk Assessment Tool (FRAX) score for fractures. Approximately 40 percent of men had baseline osteopenia (bone loss) and nine percent had baseline osteoporosis (bone loss with fracture). After being observed for 12-months of ADT treatment, it was found that 43 percent had osteopenia and 13 percent had osteoporosis. The risk of major osteoporotic fracture (3%) or hip fracture (5%) corresponded to documented FRAX score (22).

Fracture is a relatively rare adverse event of ADT; however, it can significantly impact quality of life. The pathophysiology of bone fracture in men with prostate cancer is that ADT suppresses not only 95 percent of testosterone level but also reduces approximately 80 percent of endogenous estrogen level, causing bone turnover, resorption and eventually loss in BMD (23, 34). The association between ADT and clinical fracture risk has been confirmed by several retrospective and population-based studies (6–8, 17, 24, 25). The odds ratios were slightly different for different patient populations studied. For example, use of ADT was associated with a 2.8-fold higher risk of developing any fracture risk in a New Zealand prostate cancer population (24). Whereas, the rate of fracture associated with ADT was 1.3-fold in non-metastatic prostate cancer patients and by 1.5-fold risk in metastatic patients in a United States cohort from the SEER-Medicare database (17). In a Chinese population, a single center study reported that use of ADT was associated with a 3.6-fold higher risk of having any fracture (25).

While a fracture can actually develop with any duration of ADT exposure, the fracture rate increases with cumulative ADT dose. An inverse relationship between the rate of fracture and the time since the last use of ADT has been reported (17). Radiation therapy is also reported to be associated with risk of fracture (26, 27). Other risk factors which play a role in higher risk of fracture are age, diabetes mellitus, poor performance status, and polypharmacy (7, 9, 10, 28).

Radium-223 has a four percent risk of pathological fracture or spinal cord compression as reported in the ALSYMPCA trial (29). An observational study of patients treated with six cycles of radium-223 and followed fracture rate by whole body MRI frequency at baseline, cycles 2, 4, and 1-month post-treatment (30). New fractures were commonly seen in patients treated with radium-223 monotherapy, and fracture risk was higher in patients with six or more metastatic bone sites compared to less than six sites (30). Hence, it is perhaps important to use a bone health agent with radium-223 therapy when patients have more than six bone metastases. Further studies are needed to corroborate this point.

The monoclonal antibody, denosumab, which binds to RANKL, was approved by FDA in 2011 for use in patients at high risk for fracture who are receiving ADT for non-metastatic prostate cancer to improve bone mass. However, there is no clear definition for “high risk” of fracture. Approval was based on a phase III trial which showed improvement of lumbar BMD by 5.6 percent after 24 months of denosumab therapy. This trial also showed a reduction in incidence of new vertebral fractures by 1.4% (*p* = 0.006) (31). A meta-analysis of 18 trials evaluated the benefit of using bisphosphonates in prostate cancer patients with bone metastases. No benefit was shown in overall survival. Ultimately, bisphosphonates might provide a benefit in reducing fracture risk and disease progression (32). A baseline DEXA scan before the start of ADT and a follow-up DEXA scan after 1 year of therapy is recommended by the International Society for Clinical Densitometry to monitor BMD response (33).

There are several limitations to this study. The CTEP database records bone fracture as an adverse event during its trial monitoring and would be subject to recall bias. We were interested in looking at the duration of prior ADT therapy, baseline BMD information, details of prior treatment with bone health agents, demographic data, exact duration of ADT on therapy, and other bone health data to better understand the risk of fracture with ADT exposure while being treated with investigational agents. Some of these data were not available. Men in these CTEP-sponsored trials were healthy enough to enroll in a clinical trial, and thus, passed prespecified eligibility criteria that might not represent a larger prostate cancer population. This leaves this study vulnerable to selection bias.

## Conclusions

Our study shows a very low rate of new fracture secondary to ADT-investigational agent exposure. Our observed rate might be attributed to relatively brief ADT exposure on early phase trial and short duration of clinical follow up for these trials. The concomitant use of bone health agents, especially for men with underlying co-morbidities like diabetes or conditions requiring protracted corticosteroid use, deserves further prospective study.

## Data Availability Statement

The datasets analyzed in this article are not publicly available. Requests to access the datasets should be directed to zin.myint@uky.edu.

## Author Contributions

ZM: conception and design of the study, acquisition of data, analysis, interpretation of data, drafting of the article or revising it for important content, and final approval of the version to be published. CK: conception and design of the study, acquisition of data, analysis, interpretation of data, critical review of the manuscript, and final approval of the version to be published. All authors contributed to the article and approved the submitted version.

## Conflict of Interest

The authors declare that the research was conducted in the absence of any commercial or financial relationships that could be construed as a potential conflict of interest.
